# The “McFL‐LARS” Hybrid Technique: Fascia Lata Autograft and Synthetic Reinforcement for Combined Revision Anterior Cruciate Ligament Reconstruction and Lateral Extra‐articular Tenodesis

**DOI:** 10.1002/atn2.70258

**Published:** 2026-09-11

**Authors:** Bernard de Geofroy, Adeniran Ogou, Olivier Barbier, Matthieu Peras, Paul Teixeira, Louis‐Marie Pessey, Camille Choufani

**Affiliations:** ^1^ Department of Orthopedic Surgery Hôpital Privé Clairval Marseille France; ^2^ Department of Orthopedic Surgery Sainte‐Anne Military Teaching Hospital Toulon France

## Abstract

Anterior cruciate ligament reconstruction using fascia lata autograft is a validated technique but remains limited by graft diameter or by the need to harvest a hamstring tendon. This report describes a novel hybrid approach, the “McFL‐LARS” hybrid technique, combining fascia lata autograft with a ligament advanced reinforcement system (Movmedix) synthetic ligament to enhance graft robustness and simplify harvest. A narrow strip of fascia lata is reinforced with a 6‐mm ligament advanced reinforcement system (Movmedix) ligament to produce an 8‐mm composite graft, allowing easier closure of the donor site while maintaining a strong, biologically active construct. The technique provides a simple and reproducible option for revision anterior cruciate ligament reconstruction, particularly in patients with limited autograft availability.

## Graphical abstract

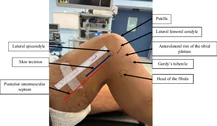

**VIDEO 1:** Patient in the supine position, operative knee prepared for arthroscopic and lateral approaches (right side): step‐by‐step summary of the complete “McFL‐LARS” hybrid technique for combined revision anterior cruciate ligament reconstruction and lateral extra‐articular tenodesis. The video shows fascia lata harvest, femoral isometric point identification, arthroscopic tunnel preparation, hybrid graft preparation with ligament advanced reinforcement system reinforcement, graft passage, fixation, and final positioning. This procedure aims to combine biologic graft incorporation, adequate graft diameter, and improved rotational control in the revision setting. Video content can be viewed at https://doi.org/10.1002/atn2.70258.

Mixed intra‐ and extra‐articular reconstructions using the fascia lata as a graft were initially developed under the impetus of David McIntosh^1^ and later refined in France by Jean Henri Jaeger.^2^ The absence of functional morbidity compared with graft harvesting from the extensor mechanism or the hamstring tendons made this technique attractive to early users.^3^ Despite satisfactory results in terms of knee stability, the invasive nature of the harvest and the complications reported at that time led to a decline in its use in favor of purely intra‐articular reconstructions using the patellar tendon, quadriceps tendon, or hamstrings.^4^ The main advantages of fascia lata harvesting are the preservation of its strong native bony insertion on Gerdy's tubercle, the ability to perform both intra‐ and extra‐articular reconstruction with a single graft, and the absence of the need for femoral fixation. Specific complications such as femoral fracture because of interference screws or tunnel widening caused by graft motion around a cortical button are thereby avoided.^5^ The fascia lata is a robust structure;^6^ however, its thinness compared with the hamstring or patellar tendons may result in a graft of small diameter. Grafts measuring less than 8 mm in diameter are associated with an increased risk of rerupture.^7^ One proposed solution is to harvest a wide strip, 4 to 5 cm in width, and roll it onto itself to achieve the desired diameter. However, this approach carries specific drawbacks, including difficulty closing the donor site, risk of postoperative hematoma, and potential for muscular herniation. Dos Santos et al.^8^ subsequently proposed reinforcing the fascia lata with the gracilis tendon. Although effective, this solution requires sacrificing a hamstring tendon, which may not be available in anterior cruciate ligament (ACL) revision cases.

To address these limitations, we describe a hybrid technique involving the harvest of a narrow strip of fascia lata reinforced with a filar ligament advanced reinforcement system (LARS) (Movmedix, Arc‐sur‐Tille, France) synthetic ligament. This hybrid technique aims to achieve an effective reconstruction with a graft of adequate diameter while facilitating closure of the fascia lata donor site.

## SURGICAL TECHNIQUE

The described technique is presented in detail in Video 1. The pearls and pitfalls of this surgical operation are listed in Table 1.

**TABLE 1:** Pearls and Pitfalls of the Technique

Surgical Steps	Pearls	Pitfalls
Harvesting the FL	Pay attention to the posterior vessels of the lateral femoral condyle	Postoperative lateral thigh hematoma
Identification of the isometric point	The lateral epicondyle and genicular vessels are useful landmarks for determining the optimal extra‐articular femoral location	Limited mobility and poor graft efficiency in case of nonisometric placement
Testing the isometric point	A tensioned suture is placed on a reference pin at Gerdy's tubercle and a point on the theoretical femoral site. During knee flexion and extension, the distance and tension of the suture should remain constant	Improper selection of isometric points leads to large variations in graft length between flexion and extension
Graft positioning	Accurate measurement of femoral tunnel length is necessary to correctly position the intra‐articular fibrillar fibers of the LARS	Incorrect positioning of the LARS may result in graft failure or rerupture
Graft fixation	Maintain the knee at 30° of flexion and the foot in neutral rotation during tibial fixation of the graft	Improper knee position during graft fixation may lead to a nonisometric reconstruction
Closure of the fascia lata	Place a few sutures to close the fascia lata after harvesting to prevent retraction	Risk of muscle herniation

FL, fascia lata; LARS, ligament advanced reinforcement system.

### Patient Positioning

**FIGURE 1:**
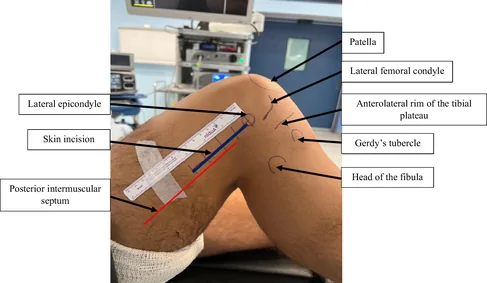
Patient in the supine position, lateral aspect of the knee (right side): preoperative skin marking of the anatomical landmarks and planned incision used for the “McFL‐LARS” hybrid technique. The lateral epicondyle, Gerdy's tubercle, fibular head, joint line, and course of the fascia lata are identified to guide safe graft harvest and reproducible lateral extra‐articular tenodesis planning.

The patient is placed in the supine position on a standard operating table, with the knee flexed to 90° (Figure 1) and stabilized using a lateral post to facilitate the arthroscopic procedure and maintain stability. The anatomical landmarks are Gerdy's tubercle, the lateral femoral epicondyle, and the posterior intermuscular septum. A tourniquet is applied to the proximal thigh, deflated during graft harvest to allow meticulous hemostasis, and inflated to 250 mm Hg during the arthroscopic phase.

### Graft Harvesting

**FIGURE 2:**
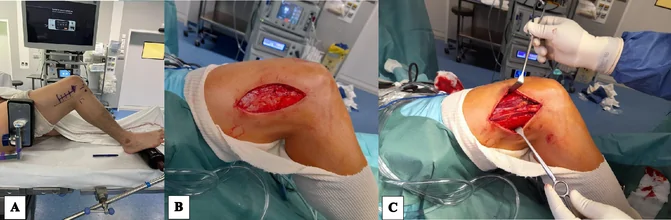
Patient in the supine position, lateral aspect of the knee (right side): surgical approach to the fascia lata through an 8‐cm longitudinal lateral incision beginning at the level of the lateral epicondyle and centered 1 cm anterior to the posterior intermuscular septum. This exposure allows controlled harvest of the fascia lata autograft while preserving surrounding lateral structures. (A) Supine position with the knee flexed at 90°. (B) Skin incision down to the FL, with subcutaneous tissue dissection to allow greater flexibility of the FL during closure. (C) Incision of a 2‐cm‐wide and 13‐cm‐long strip of FL starting from the lateral epicondyle. (FL, fascia lata.)

**FIGURE 3:**
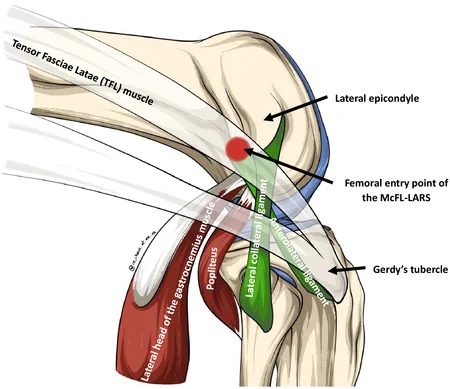
Patient in the supine position, lateral view of the right knee: extra‐articular identification of the femoral isometric point, corresponding to the femoral entry site for the combined “McFL‐LARS” graft on the lateral aspect of the distal femur (red marker). Accurate localization of this point is essential to achieve appropriate graft isometry during knee flexion‐extension and to optimize the tenodesis effect without overconstraint. (TFL, tensor fasciae latae.)

For fascia lata harvesting, a posterolateral thigh incision is made from the lateral femoral epicondyle extending proximally for 8 cm. The subcutaneous tissue is dissected along the incision, extending anteriorly to Gerdy's tubercle and proximally up to 13 cm above the lateral epicondyle, corresponding to the desired intra‐articular graft length. The fascia lata is exposed using 2 long‐handled retractors (Langenbeck type) (Figure 2). A strip of fascia lata is harvested up to Gerdy's tubercle while preserving its bony insertion. The graft measures approximately 13 cm in length from the lateral epicondyle proximally, with a width of 2, and 1 cm in width from the epicondyle to Gerdy's tubercle, corresponding to the extra‐articular tenodesis portion. The posterior incision of the fascia lata is made 1 cm anterior and parallel to the posterior intermuscular septum. The distal end of the strip is grasped with a clamp and dissected in a craniocaudal direction from the underlying vastus lateralis muscle and articular capsule. Once the harvest is completed, the proximal portion of the fascia lata is partially closed with interrupted figure‐of‐8 sutures using No. 2 braided absorbable synthetic suture to prevent retraction. The femoral isometric point, where the outside‐in femoral tunnel will be created, is then identified. With the knee flexed at 90°, this point lies 1 cm posterior to the lateral collateral ligament and 1 cm distal to the lateral femoral epicondyle, within a fatty triangle overlying the vessels of Lemaire. The use of a compass or a taut suture over a guide pin placed just posterior to Gerdy's tubercle helps verify isometry by positioning 1 tip on Gerdy's tubercle and the other on the theoretical femoral point. During knee flexion and extension, the length and tension of the suture should remain constant, ensuring that the graft remains taut in both positions (Figure 3). This entire step is performed without the tourniquet inflated to allow meticulous hemostasis and minimize the risk of postoperative hematoma.

### Arthroscopic Phase

The arthroscopic phase is performed under tourniquet control, inflated to 250 mm Hg after venous exsanguination. Standard portals are used: anterolateral for visualization and anteromedial for instrumentation. All necessary procedures are carried out, including notch preparation, meniscal repair or resection as indicated, creation of the femoral tunnel using an outside‐in guide from the previously identified isometric point, and drilling of the tibial tunnel with its intra‐articular entry point located between the tibial spines, ensuring no impingement with the femur in extension. Both bone tunnels are reamed to 8 mm in diameter (the LARS (Movmedix) ligament used measures 6 mm, and when enveloped with the fascia lata, the composite graft reaches a final diameter of approximately 8 mm).

### Preparation of the “McFL‐LARS” Hybrid Graft

**FIGURE 4:**
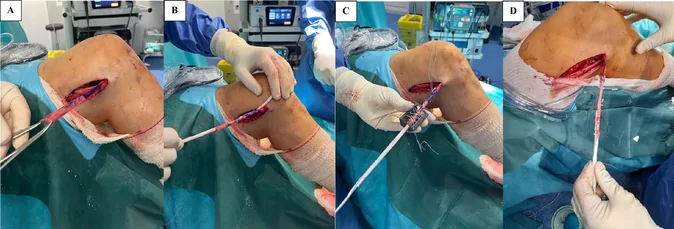
Back‐table view of graft preparation: preparation of the hybrid “McFL‐LARS” construct combining a fascia lata autograft with synthetic LARS reinforcement. This hybrid configuration is intended to provide biologic incorporation from the autograft together with immediate mechanical reinforcement for combined revision anterior cruciate ligament reconstruction and lateral extra‐articular tenodesis. (A) The FL strip is detached proximally; it measures 13 cm in length from the lateral epicondyle and 2 cm in width, allowing it to cover the 6‐mm‐diameter LARS (circumference 18.8 mm). The blue‐shaded area represents the intra‐articular portion corresponding to the fibrillar zone of the LARS. (B) The LARS is placed inside the iliotibial band on a right knee, and the assembly is secured with initial Vicryl 0 sutures. (C) Tubularization of each graft end using the SpeedTrap DePuy Synthes system. (D) Final appearance of the graft after preparation. (FL, fascia lata; LARS, ligament advanced reinforcement system.)

**FIGURE 5:**
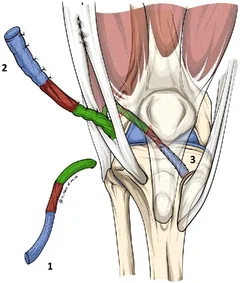
Anterior view of the right knee, schematic illustration: 3‐step diagram showing graft preparation, passage, and final placement of the “McFL‐LARS” hybrid construct. This figure summarizes the key technical sequence for combined revision anterior cruciate ligament reconstruction and lateral extra‐articular tenodesis, highlighting how the hybrid graft addresses both intra‐articular stability and lateral rotational control. (blue: intratibial graft; red: intra‐articular graft; green: intrafemorally graft).

Several models of LARS ligaments (Movmedix, Arc‐sur‐Tille, France) are compatible with this technique. A model featuring a central filar segment flanked by 2 solid sections (such as the PC 60 or AC 80 models) should be used. The length of the femoral tunnel is then measured and transposed onto the harvested fascia lata to determine the fixation point of the LARS on the graft. The goal is to position a solid portion of the LARS within the femoral tunnel, followed by the filar segment intra‐articularly, and then another solid portion within the tibial tunnel. The fascia lata strip is unfolded and tensioned with 2 clamps. The LARS (Movmedix) ligament is positioned inside the fascia lata at the appropriate distance so that its free filar fibers will lie intra‐articularly (Figures 4 and 5). The construct is then tubularized at both ends using the SpeedTrap system (DePuy Synthes Mitek, Johnson & Johnson, New Zealand). The graft is calibrated to determine its diameter at both the femoral and tibial ends, which is typically 8 mm; if greater, the femoral and tibial tunnels are adjusted accordingly. The composite graft is then soaked in vancomycin solution to reduce the risk of infection.

The graft is then passed superficial to the LCL from the femur to the tibia using a shuttle suture system. Repeated cycling of the knee is performed to optimize graft tensioning. The knee is positioned at 30° of flexion with the foot in neutral rotation. Tibial fixation is achieved using a bioabsorbable interference screw, with diameter and length adapted to the patient's sex, morphology, and bone quality, typically 1 mm larger than the graft diameter in men and 2 mm larger in women. Femoral fixation is generally unnecessary but may be performed using a same‐size interference screw if desired. The fascia lata donor site is closed progressively with interrupted sutures, ensuring the absence of muscular herniation. Local anesthetic infiltration is performed intraoperatively, particularly at the fascia lata harvest site, as part of the multimodal pain management protocol. The advantages and disadvantages of this surgical technique are listed in Table 2.

**TABLE 2:** Advantages and Disadvantages of the Technique

Advantages	Disadvantages
Preservation of the extensor mechanism and hamstrings	Longer operative time
Preservation of the strong natural attachment of the graft on Gerdy's tubercle	8‐cm lateral incision
Fixation of the graft only to the tibia	Learning curve
Harvesting of a narrower and shorter fascia lata strip	Technically demanding procedure
Easier closure of the fascia lata	Risk of hematoma or muscular hernia
Simultaneous reconstruction of the anterior cruciate ligament and the anterolateral ligament with a single graft	Automatically associated lateral tenodesis

## DISCUSSION

Revision ACL reconstruction is challenging, especially when autologous tissue is limited. The McFL‐LARS hybrid technique, which combines fascia lata autograft with synthetic reinforcement (such as LARS), aims to address graft availability and improve outcomes. Research on this specific hybrid approach is limited, but related studies on autografts, synthetics, and their combinations provide insight. Recent systematic reviews show that synthetic ligaments like LARS can restore knee stability and function as effectively as autografts, with some studies reporting better patient‐reported outcomes and fewer complications in the short term. Some studies report improved early rehabilitation and stability with synthetics, but long‐term superiority is unproven.^9^, ^10^, ^11^, ^12^ Although synthetic grafts (LARS) offer advantages such as faster recovery and less donor‐site morbidity, they are associated with a higher rate of revision surgeries over time. Most publications addressing the use of synthetic ligaments are based on early series of ACL reconstructions. After a surge in popularity during the 1980s,^13^ numerous failures and complications led to a decline in their use for this indication.^14^, ^15^ The causes of failure were multiple, with an unacceptably high rupture rate. Design flaws promoted excessive abrasion because of shear forces between crossed fibers and along the edges of the bone tunnels, resulting in carbon debris and graft rupture. In addition, these devices exhibited inadequate mechanical properties—being overly rigid, with poor resistance to torsion and elongation—and a lack of biocompatibility.^15^

Over the past 15 years, the development of more biocompatible materials and a better understanding of knee kinematics have led to a new generation of synthetic ligaments.^16^ The LARS ligament, composed of polyethylene terephthalate fibers, features a dual‐structure design: an intraosseous portion with longitudinal fibers interconnected by transverse knitted links and an intra‐articular portion with free, parallel fibers oriented at 30°. This configuration mimics the natural orientation of collagen fibers in the native ligament and represents the main innovation of the LARS. The alternating load distribution between fibers during flexion and extension reduces shear forces, while the porous architecture of the intra‐articular segment promotes tissue ingrowth. The LARS can be used either as a primary reconstruction or as reinforcement to an existing graft. It is more widely used internationally than in France, where the *French National Authority for Health* has approved its indication for ACL and PCL revision surgery when all autograft options have been exhausted and exceptionally for multiligament knee injuries when insufficient autologous tissue is available.

Today, autografts are widely used as the first‐line option but present certain limitations, including a risk of rerupture ranging from 2.8% to 28% in high‐risk patients,^17^ donor‐site morbidity,^18^ and limited availability in multiligament injuries.^19^ Allografts represent an interesting alternative in such cases but are associated with rupture rates of up to 20%,^20^ lack of standardized size and mechanical characteristics, and complex, costly logistics.^21^

The original fascia lata ACL reconstruction technique is an alternative that offers several advantages, including the ability to perform a combined intra‐ and extra‐articular reconstruction, absence of morbidity to the extensor mechanism, and preservation of the hamstring tendons, which contribute to anterior drawer control in flexion. However, its main limitation is that the graft diameter is often less than 8 mm, which is associated with a higher risk of rerupture.^22^

Hence, the rationale for this technique in revision ACL reconstruction is to combine the advantages of fascia lata harvesting with those of the LARS synthetic ligament. Direct evidence for the McFL‐LARS hybrid in ACL revision is lacking, but similar hybrid approaches in other joints (rotator cuff repair) show that combining fascia lata autograft with LARS can improve functional outcomes and graft healing rates compared with other synthetic scaffolds.^23^ This suggests potential benefits for hybrid use in ACL revision, though more targeted research is needed.

Fascia lata harvesting and closure are simplified because a smaller strip is required compared with the original technique. The addition of a 6.5‐mm LARS ligament requires only a 2‐cm‐wide fascia lata strip to obtain a graft of sufficient size. The fascia lata envelops the LARS, which increases the overall graft diameter by approximately 2 mm. The resulting 8‐mm graft diameter is comparable to that achieved with standard ACL reconstruction techniques.^12^, ^14^ The combination of a biological and synthetic graft may also promote the biological integration of the LARS fibers.

This technique is reproducible and easy to perform, even for surgeons who are not initially familiar with fascia lata harvesting for ACL reconstruction. At 1‐year follow‐up in 14 patients, no complications were observed except for 1 postoperative hematoma requiring surgical drainage (no graft intolerance, impingement, or rerupture). Follow‐up is ongoing to evaluate return‐to‐sport outcomes and long‐term functional results. As described in the original McFL technique, this approach maintains the advantage of performing a concomitant lateral tenodesis, thereby providing improved rotational control.^24^

The McFL‐LARS hybrid technique appears to be a simple, reproducible, and biologically advantageous option for revision ACL reconstruction when autograft options are limited. It combines the biological integration of fascia lata with the mechanical strength of synthetic reinforcement, providing a graft of sufficient diameter with reduced donor‐site morbidity and the potential for improved rotational control.

## DISCLOSURES

The authors (O.B., C.C.) declare the following financial interests/personal relationships which may be considered as potential competing interests: O.B. reports consulting fees from Xnox, FH, and Arthrex. C.C. reports consulting fees from Dedienne. The other authors (B.G., A.O., M.P., P.T., L‐M.P.) declare that they have no known competing financial interests or personal relationships that could have appeared to influence the work reported in this article.
